# B7-H3 promotes gastric cancer cell migration and invasion

**DOI:** 10.18632/oncotarget.17847

**Published:** 2017-05-13

**Authors:** Yecheng Li, Xiaodong Yang, Yong Wu, Kui Zhao, Zhenyu Ye, Junjia Zhu, Xiaohui Xu, Xin Zhao, Chungen Xing

**Affiliations:** ^1^ Department of General Surgery, Second Affiliated Hospital of Soochow University, Suzhou 215004, Jiangsu, P. R. China; ^2^ Department of General Surgery, First Affiliated Hospital of Soochow University, Suzhou, 215006, P.R. China

**Keywords:** gastric cancer, B7-H3, CXCR4, migration, invasion

## Abstract

B7-H3 (B7 homologue 3, CD276) is a member of the B7 immunoregulatory family and promotes tumor progression. The present study demonstrated that B7-H3 promotes gastric cancer cell migration and invasion. shRNA-mediated B7-H3 silencing in the N87 gastric cancer cell line suppressed cell migration and invasion *in vitro* and *in vivo*; downregulated metastasis-associated CXCR4; and inhibited AKT, ERK, and Jak2/Stat3 phosphorylation. B7-H3-silenced cells injected into the tail veins of 4-week-old female BALB/c nude mice produced fewer metastases than control cells, and resulted in longer survival times. Immunofluorescence analyses confirmed B7-H3/CXCR4 colocalization in N87 cells, and co-immunoprecipitation assays showed a direct interaction between the two proteins. Our analysis of 120 tissue samples from gastric cancer patients showed that increased B7-H3 expression correlated positively with both tumor infiltration depth and CXCR4 expression. These findings suggest that B7-H3 and CXCR4 may be novel targets for anti-gastric cancer therapeutics.

## INTRODUCTION

Gastric cancer is the fourth most frequently diagnosed cancer and the second most common cause of cancer-related deaths worldwide [1]. Improved diagnostic and therapeutic strategies have improved early-stage gastric cancer detection and decreased patient mortality [2]. Still, although new anticancer agents, such as S-1, tananes, capecitabine, oxaliplatin, and irinotecan [3–7], have improved gastric cancer patient prognoses, survival rates remain unsatisfactory [8, 9]. Novel biomarkers are needed to improve early tumor metastasis predictions, and effective anti-metastasis/invasion agents are required to enhance patient outcomes.

B7-H3 (B7 homologue 3, CD276), a B7 immunoregulatory family member, is a type I transmembrane protein with an immunoglobulin-like structure [10]. There are two B7-H3 isoforms: murine 2Ig B7-H3 and human 4Ig B7-H3 [11–13]. The role of the protein in the adaptive immune response remains controversial [14]. B7-H3 reportedly both activates and inhibits T cell responses [15, 16], and may also promote disease progression in pancreatic carcinoma [17], hepatocellular cancer [18], human esophageal cancer [19], non-small cell lung cancer [20], prostate cancer [21], endometrial cancer [22], and gastric cancer [2]. However, B7-H3 studies in gastric cancer produced conflicting results [23], and the B7-H3 mechanism of action in malignant tumors remains unclear.

Metastasis is a complex process involving cell intravasation to vessels, extravasation, invasion into a distant organ, and establishment and proliferation of secondary tumors in a new microenvironment [24, 25]. Many molecules are implicated in the process of cancer metastasis. CXCR4 is a stromal cell-derived factor-1 (SDF-1) receptor that reportedly promotes cancer progression, including cell migration, invasion, and seeding to distal tissues [25–27], and is abnormally over-expressed in gastric cancer tissues [28]. In addition, AKT, ERK, and Jak2/Stat3 signaling activation is associated with metastasis [29, 30].

Here, we showed that B7-H3 promotes gastric cancer cell migration and invasion, and its upregulation enhances tumor infiltration depth. B7-H3 silencing downregulated CXCR4 expression and inhibited phosphorylation of AKT, ERK, and Jak2/Stat3 pathway members. Finally, we confirmed that B7-H3 and CXCR4 colocalize in gastric cancer cells and can interact directly.

## RESULTS

### B7-H3 and CXCR4 expression in gastric cancer tissue samples

**Figure 1:**
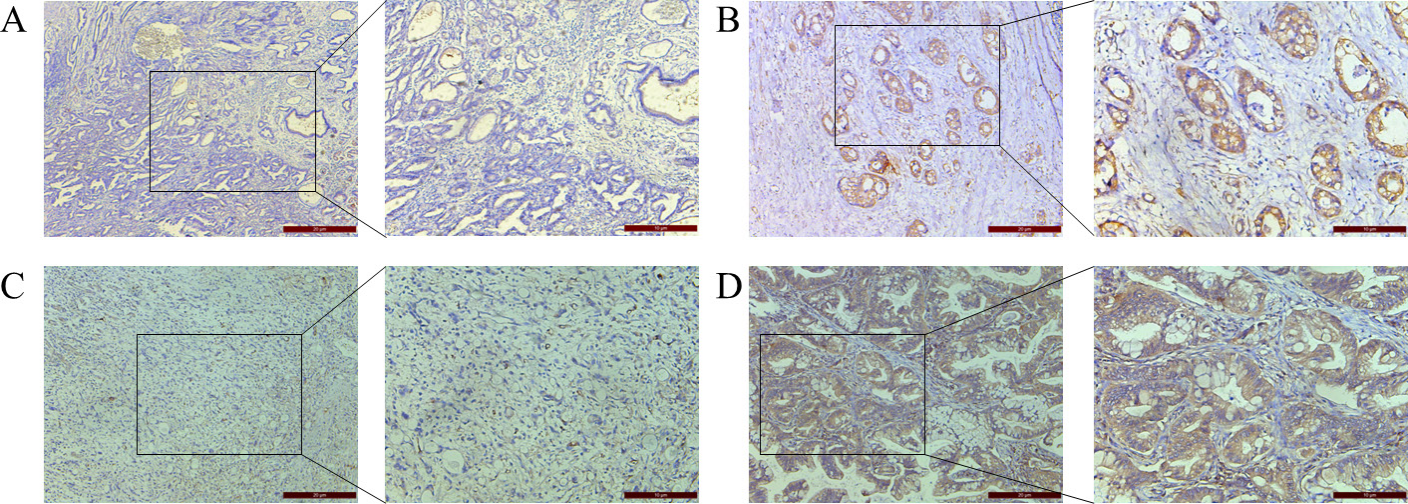
IHC staining of gastric cancer patient tissue samples B7-H3 negative (**A**) and positive (**B**) and CXCR4 negative (**C**) and positive (**D**) expression in gastric tissue. Magnification: 100× left, 200× right.

Immunohistochemical (IHC) staining showed that B7-H3 was expressed in gastric carcinoma cell membranes and cytoplasm, and was observed in 69.2% of tissue samples (Figure 1A–1B). B7-H3 expression in gastric cancer was not correlated with patient age, sex, lymph node metastasis, degree of differentiation, or HER-2 status. However, B7-H3 levels in cancer tissues were positively correlated with tumor infiltration depth (*P* = 0.005; Table 1). B7-H3 expression was correlated with CXCR4 expression (*P* < 0.001; Figure 1C–1D, Table 2).

**Table 1:** Correlation between tumor B7-H3 expression and pathologic features of gastric cancer patients

Variables	All cases	B7-H3 expression		*P*
		Negative	Positive	
Gastric cancer	120	37 (30.8%)	83 (69.2%)	
Age				
≤ 50	17	7 (41.2%)	10 (58.8%)	*P* = 0.319
> 50	103	30 (29.1%)	73 (70.9%)	
Gender				
male	87	25(28.7%)	62 (71.3%)	*P* = 0.419
female	33	12 (36.4%)	21 (63.6%)	
infiltration depth of tumor				
infiltration at mucosa	9	7 (77.8%)	2 (22.2%)	*P* = 0.005
infiltration at shallow muscularis	18	6 (33.3%)	12 (66.7%)	
infiltration at deep muscularis	93	24 (25.8%)	69 (74.2%)	
LN metastasis				
No	58	20 (34.5%)	38 (65.5%)	*P* = 0.402
Yes	62	17 (27.4%)	45 (72.6%)	
degree of differentiation				
well-differentiated	3	1 (33.3%)	2 (66.7%)	*P* = 0.093
moderately-differentiated	50	10 (20.0%)	40 (80.0%)	
poorly-differentiated	67	26 (38.8%)	41 (61.2%)	
HER2				
Positive	96	32 (33.3%)	64 (66.7%)	*P* = 0.239
Negative	24	5 (20.8%)	19 (79.2%)	

Values in bold signify *P* > 0.05; b. Chi-square test for trend.

**Table 2:** Correlation between tumor B7-H3 expression and CXCR4 expression of gastric cancer patients

CXCR4 expression	B7-H3 expression		*P*
	Negative	Positive	
Negative (*n =* 51)	29 (56.9%)	22(43.1%)	***P* < 0.001**
Positive (*n =* 69)	8 (11.6%)	61(88.4%)	

a. Values in bold signify P < 0.05; b. Chi-square test for trend.

### shRNA-mediated B7-H3 silencing in N87 gastric cancer cells

**Figure 2:**
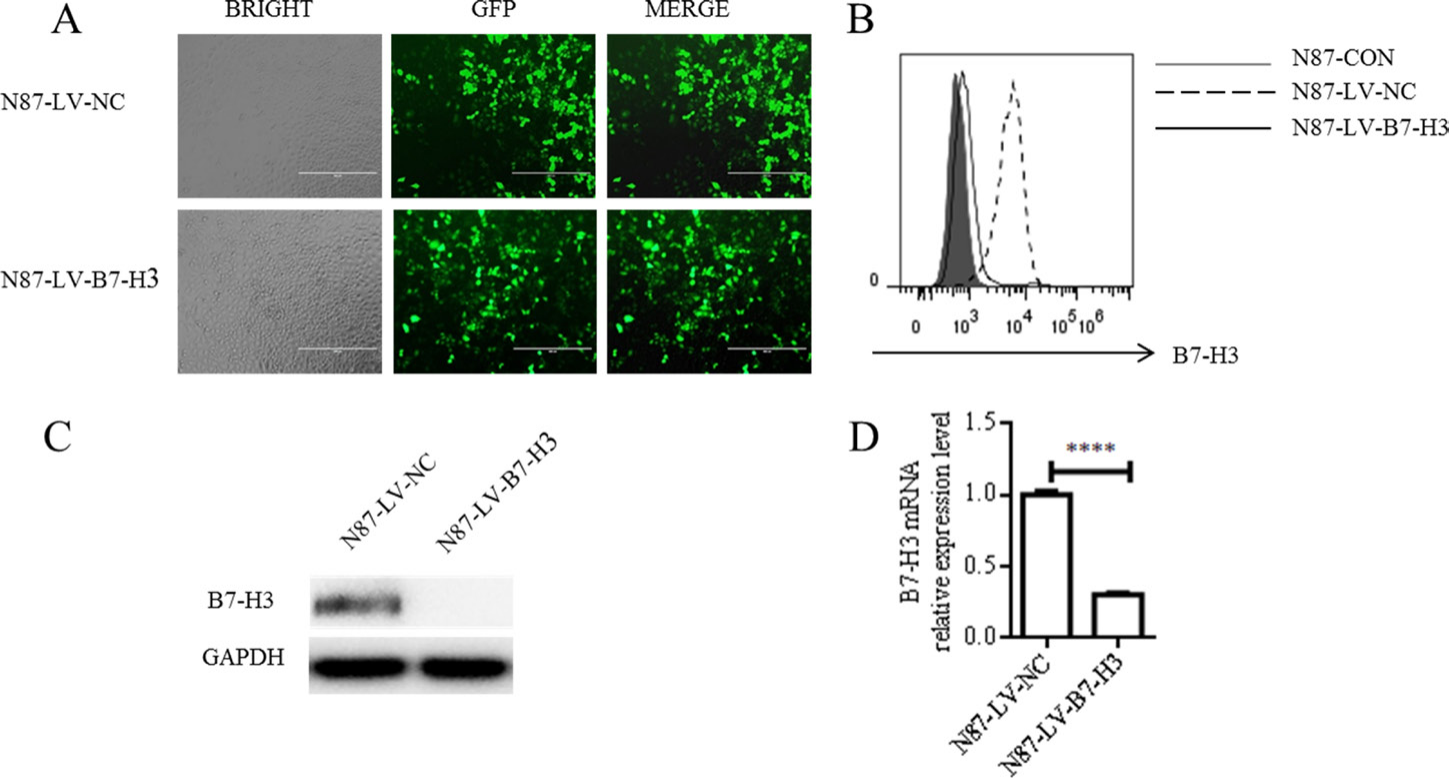
shRNA-mediated B7-H3 silencing N87 cells were infected with either LV-NC or LV-B7-H3 (**A**) GFP detection via fluorescence microscopy showed high infection efficiency (Magnification, 100×). B7-H3 membrane protein as shown by flow cytometry (**B**) B7-H3 plasma protein as shown by western blotting (**C**) B7-H3 mRNA levels as shown by RT-PCR (**D**) ****P < 0.0001.

Fluorescence microscopy showed high lentiviral infection efficiency in treated N87 cells (Figure 2A). We used flow cytometry, western blotting, and real-time PCR to analyze B7-H3 expression in both LV-NC- and LV-B7-H3-infected N87 cells. LV-B7-H3-infected cells exhibited lower B7-H3 membrane and plasma protein levels than LV-NC-infected cells (Figure 2B–2C). shRNA-mediated silencing reduced membrane protein levels by 88.03% and plasma protein levels by 98.94%. Band densitometric analyses by Image J showed lower B7-H3 plasma protein levels in LV-B7-H3-infected cells than in LV-NC-infected cells (*P* < 0.0001; Supplementary Figure 1A). mRNA levels exhibited a similar pattern (mean 0.302 ± 0.013, *P* < 0.0001; Figure 2D).

### B7-H3 silencing reduced N87 cell migration and invasion

**Figure 3:**
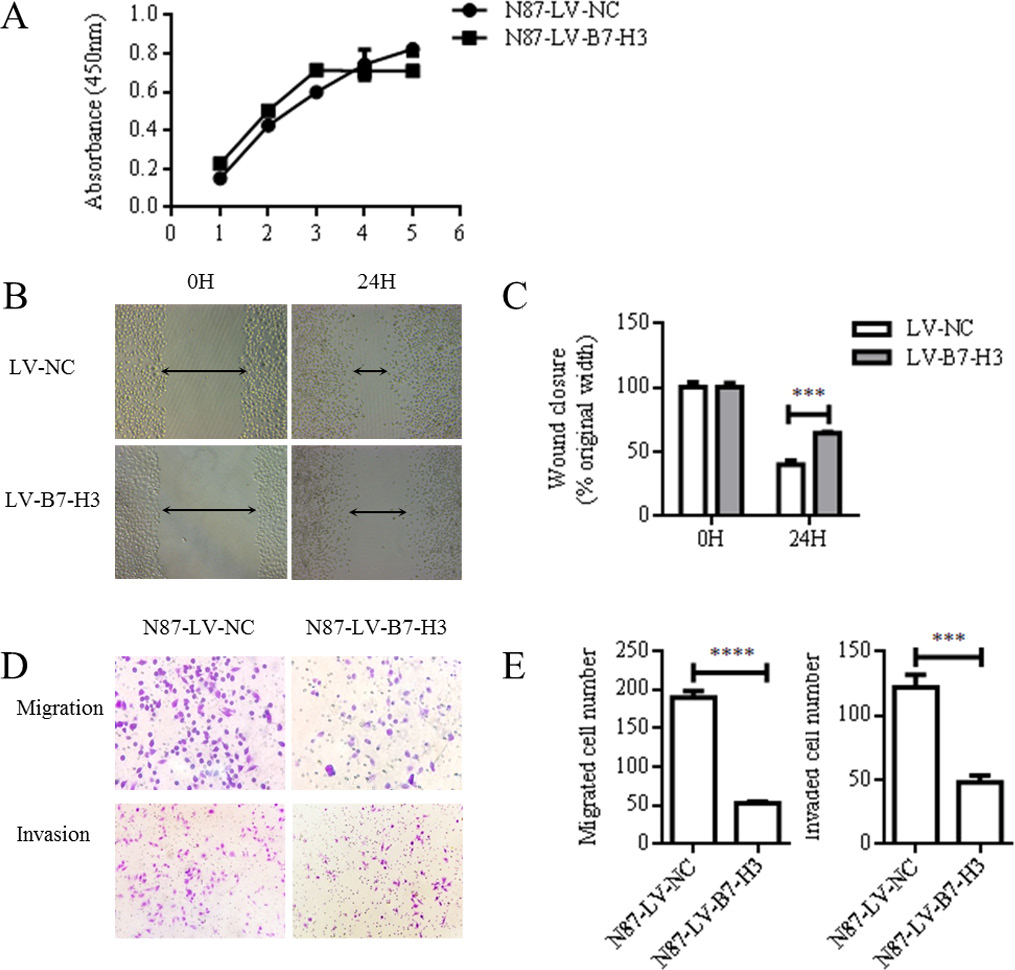
B7-H3 silencing reduced N87 cell invasion and migration CCK8 assay results showed no difference between LV-NC- and LV-B7-H3-infected N87 cells (*P* > 0.05) (**A**) Representative images of LV-NC- and LV-B7-H3-infected N87 cell migration in the wound healing assay at 0 and 24 h (Magnification, 100×) (**B**) The monolayer was re-established more quickly in control cells than in B7-H3-silenced cells (**C**) Cell migration and invasion as detected by transwell assay (**D**) Representative images of invading cells (Magnification, 200×). Numbers of invading cells are expressed as means ± SD of three independent experiments (**E**) ***P < 0.0005, ****P < 0.0001.

CCK-8 assay results showed no difference between LV-NC- and LV-B7-H3-infected N87 cell proliferation rates (*P* > 0.05; Figure 3A). However, wound healing assay results showed that wound healing was reduced in B7-H3-silenced N87 cells compared to controls (mean 39.63 ± 1.918% versus 64.11 ± 0.6549%, *P* < 0.0005; Figure 3B–3C). Similarly, LV-B7-H3-infected N87 cells migrated less in a transwell assay after 24 h compared to controls (mean 52.33 ± 1.202 versus 189.7 ± 4.910 per three random microscopic fields, reduction of 72.415%, *P* < 0.0001; Figure 3D–E). B7-H3 silencing also reduced N87 cell invasion by 60.38% compared to controls (mean 47.67 ± 3.180 versus 121.7 ± 5.812 per three random microscopic fields, *P* < 0.0005) (Figure 3D–3E).

### B7-H3 silencing inhibited N87 cell lung metastasis in nude mice

**Figure 4:**
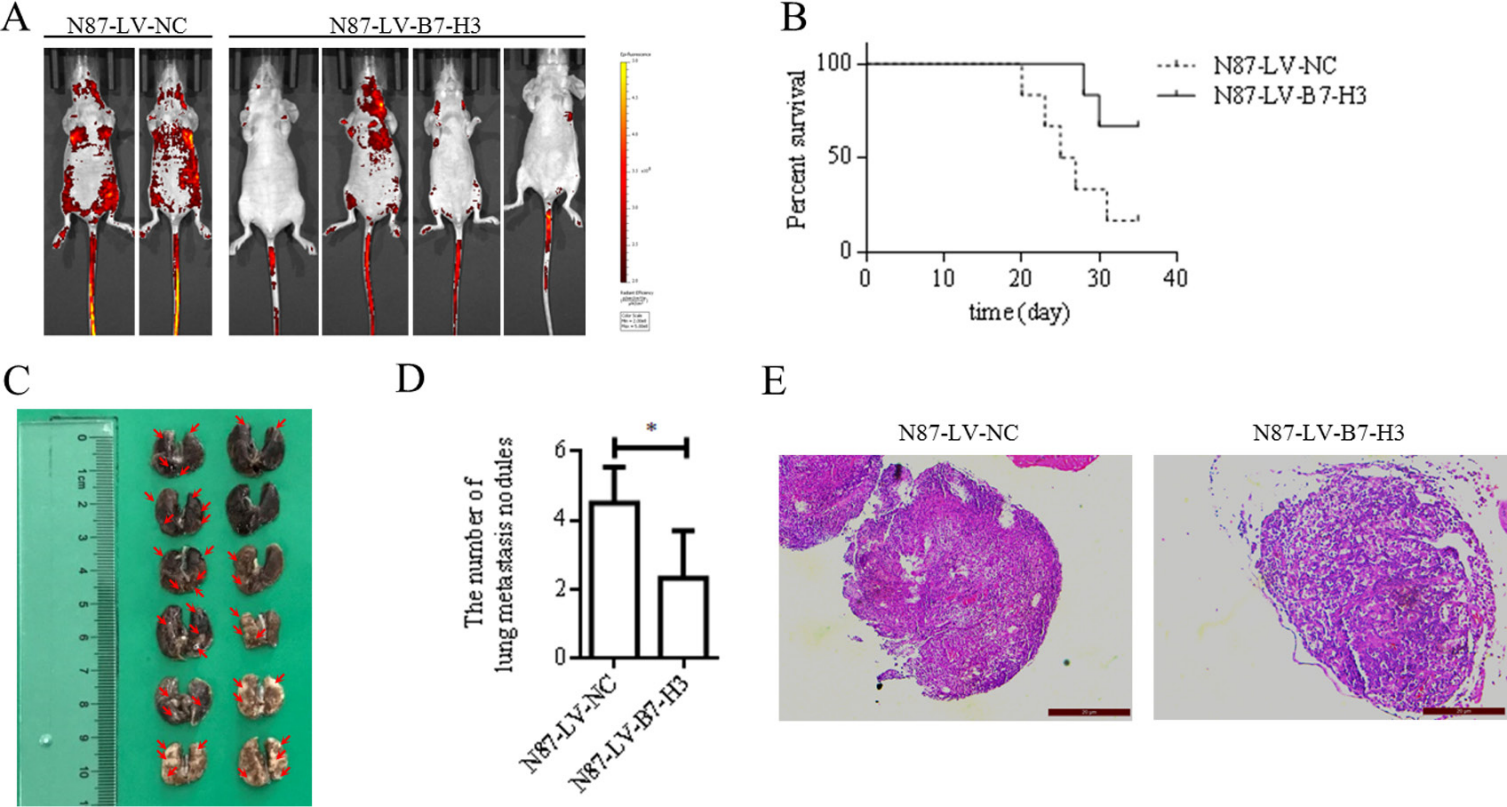
B7-H3 silencing reduced N87 cell lung metastasis in nude mice (*n* = 6 per group) (**A**) *In vivo* imaging analysis in mice four weeks after injection with LV-NC- or LV-B7-H3-infected N87 cells (**B**) Kaplan-Meier plots showing survival time for LV-NC and LV-B7-H3 mice (**C**) Representative photographs of lung metastases in LV-NC and LV-B7-H3 mice (red arrows) (**D**). Mice injected with LV-B7-H3-infected N87 cells had fewer metastases than controls *P < 0.05. (**E**) H&E staining of mouse lung metastases E. Magnification, ×200.

LV-B7-H3- or LV-NC-infected N87 cells were injected into nude mouse tail veins (*n* = 6 mice per group). Four weeks after injection, mice were imaged to detect green fluorescent protein-positive (GFP^+^) cells in organs. Mice injected with LV-B7-H3-infected N87 cells had fewer metastases than controls (Figure 4A).

Mouse survival was analyzed using the Kaplan-Meier method (Figure 4B). Five of six mice injected with LV-NC-infected cells were sacrificed due to apparent symptoms of metastatic disease, as compared to only two of six in the LV-B7-H3 group (Gehan-Breslow-Wilcoxon test, *P* < 0.05).

Mice were euthanized at five weeks, and tissues were observed under a dissecting microscope. The lung was the predominant organ with metastases (Table 3). Mice injected with LV-B7-H3-infected N87 cells had more lung metastases than mice injected with LV-NC-infected cells (mean 4.500±0.4282 versus 2.333±0.5578, *P* < 0.05; Figure 4C–4D).

**Table 3:** Metastasis pattern in nude mice injected with N87 LV-NC group and LV-B7-H3 group cells

Organ	LV-NC group		LV-B7-H3 group	
Lung	6/6	100%	5/6	83%
Liver	0/6	0%	0/6	0%
Spleen	1/6	17%	0/6	0%
Renal	0/6	0%	0/6	0%
Heart	0/6	0%	0/6	0%

The number of samples with detectable metastasis from N87 LV-NC group and LV-B7-H3 group was recorded.

Mouse lung and spleen tissues were analyzed via H&E staining to further assess metastases. No major differences in lung metastasis morphology were observed between B7-H3-silenced and control cell-treated mice (Figure 4E). H&E staining of spleen metastases is shown in Supplementary Figure 1C.

### Effect of B7-H3 silencing on metastasis-associated molecules

**Figure 5:**
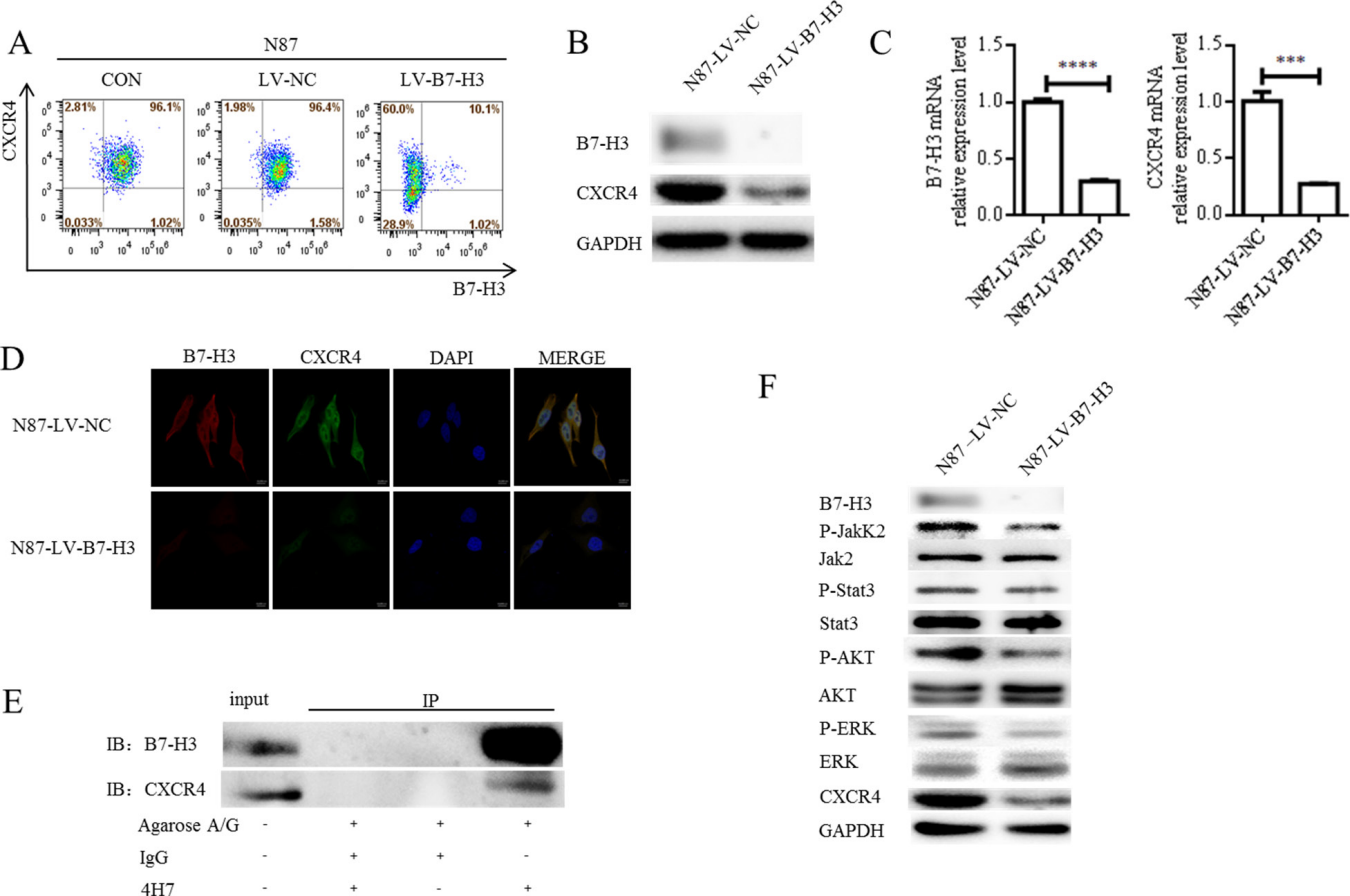
Effects of B7-H3 silencing on metastasis-associated molecules B7-H3 and CXCR4 membrane protein levels in LV-NC- and LV-B7-H3-infected N87 cells as shown by flow cytometry (**A**) B7-H3 and CXCR4 plasma protein levels as shown by western blotting (**B**) CXCR4 was downregulated in B7-H3-silenced cells. B7-H3 and CXCR4 mRNA levels as shown by RT-PCR in LV-NC- and LV-B7-H3-infected N87 cells (**C**) ****P < 0.0001, ***P < 0.0005. B7-H3 and CXCR4 colocalization was observed in the cell membrane and cytoplasm via IF confocal microscopy (**D**) Colocalization of B7-H3 (red fluorescence) and CXCR4 (green fluorescence) was observed in the merged image (yellow fluorescence). Nuclei were counterstained with DAPI (blue fluorescence) (scale bar 10,000 nm). CXCR4 interacts with B7-H3 in LV-NC-infected N87 cells (**E**) CXCR4 was pulled down with B7-H3 using an anti-B7-H3 antibody, whereas neither protein was recovered when a control antibody (IgG) or no antibody was used for immunoprecipitation. B7-H3 silencing in N87 cells reduced AKT, ERK, Jak2, and Stat3 phosphorylation as shown by western blotting (**F**)

B7-H3 silencing downregulated the metastasis-associated molecule, CXCR4, in N87 cells, as confirmed by flow cytometry, western blotting, and real-time PCR (Figure 5A–5C Supplementary Figure 1A–1B). B7-H3 and CXCR4 colocalization was examined in LV-B7-H3- and LV-NC-infected N87 cells via immunofluorescence (IF) confocal microscopy. Colocalization (yellow fluorescence) was assessed by superimposing B7-H3 (red fluorescence) and CXCR4 (green fluorescence) confocal images, and suppressing all colors except yellow. Nuclei were counterstained with DAPI. Fluorescence imaging demonstrated a high degree of B7-H3 localization with CXCR4 in the cell membrane and cytoplasm (Figure 5D). In B7-H3-silenced N87 cells, CXCR4 fluorescence intensity was reduced.

To assess the possibility of a direct interaction between B7-H3 and CXCR4 in N87 cells, total protein extracted from LV-NC-infected N87 cells was immunoprecipitated with an anti-B7-H3 antibody (4H7). Precipitated proteins were analyzed by immunoblotting with either B7-H3 or CXCR4 antibodies. CXCR4 was pulled down with B7-H3 by the anti-B7-H3 antibody, whereas neither protein was recovered when a control antibody (IgG) or no antibody was used. Co-immunoprecipitation with each specific antibody showed an association between B7-H3 and CXCR4 in N87 cells (Figure 5E).

AKT, ERK, and Jak2/Stat3 signaling aberrations are known to promote metastasis [29, 30]. We found that B7-H3 silencing in N87 cells reduced AKT, ERK, Jak2, and Stat3 phosphorylation as shown by western blotting (Figure 5F and Supplementary Figure 1D). This suggests that B7-H3 silencing suppressed gastric cancer cell migration and invasion by reducing AKT, ERK, and Jak2/Stat3 pathway activation.

## DISCUSSION

B7-H3 is a type I transmembrane protein that shares 20–27% amino acid identity with other B7 family members [10, 16]. B7-H3 was first reported to stimulate CD4^+^ and CD8^+^ T cells to increase cytotoxic T lymphocyte activity [31]. However, B7-H3 was recently implicated as a potent inhibitor of T cell activity [12, 15]. B7-H3 not only regulates the T cell-mediated immune response, but also may promote cancer progression. Zhao, *et al*. reported that B7-H3 downregulation in human pancreatic carcinoma cells inhibited cell migration and invasion, but had no apparent impact on cell proliferation [17]. In human hepatocellular cancer, B7-H3 promoted cell proliferation, adhesion, migration, and invasion [18]. Chen, *et al*. associated B7-H3 expression with esophageal cancer cell proliferation, colony formation, migration, and invasion [19]. Kang, *et al*. found that the epithelial-mesenchymal transition (EMT) induced by B7-H3, partially via Jak2/STAT3/Slug signaling, underlies hepatocellular carcinoma development and metastasis [32]. Still, the B7-H3 mechanism of action in malignant tumors remains unclear.

The present study retrospectively studied B7-H3 in 120 human gastric cancer cases. Our results showed that higher B7-H3 levels in gastric cancer tissues was associated with tumor infiltration depth, in agreement with the findings of Wu, *et al* [23]. We also investigated the impacts of shRNA-mediated B7-H3 silencing in the gastric cancer cell line, N87. CCK-8 assay results showed no difference between B7-H3-silenced N87 cells and controls (*P*>0.05). However, wound healing and transwell assays showed that B7-H3 silencing reduced N87 cell migration and invasion. B7-H3-silenced cells injected into nude mice produced fewer metastases than control cells, and led to longer survival times than controls.

CXCR4 is a stromal cell derived factor-1 (SDF-1) receptor involved in cancer cell migration and invasion [25–27]. CXCR4 was downregulated in B7-H3-silenced N87 cells, as shown by flow cytometry, western blotting, and real-time PCR. We confirmed B7-H3 and CXCR4 colocalization via IF imaging, and direct interaction through a co-immunoprecipitation study. B7-H3 silencing in N87 cells also inhibited AKT, ERK, and Jak2/Stat3 phosphorylation. B7-H3 likely works in concert with these molecules and pathways to promote gastric cancer cell invasion and metastasis.

Ours is the first report that B7-H3 silencing downregulates CXCR4. B7-H3 activation of AKT, Jak2, and Stat3, but not ERK, was reported previously [32–36]. Recent studies reported that CXCR4 induced cancer cell migration and invasion by activating AKT, ERK, Jak2, and Stat3 [37–40]. B7-H3 may activate AKT, ERK, and Jak2/Stat3 signaling through CXCR4, although this requires further study.

In summary, we investigated the role of B7-H3 in gastric cancer cell migration and invasion *in vitro* and *in vivo.* We showed that B7-H3 silencing downregulated CXCR4 and inhibited AKT, ERK, and Jak2/Stat3 phosphorylation. We confirmed B7-H3/CXCR4 colocalization and direct interaction. Our findings suggest that B7-H3 and/or its associated molecules, including CXCR4, may be novel targets for anti-gastric cancer therapeutics.

## MATERIALS AND METHODS

### Reagents

The following primary antibodies (anti-human) were used: AKT, phospho-AKT, MAPK(ERK1/2), phospho-MAPK(ERK1/2), Jak2, phospho-Jak2, Stat3, and phospho-Stat3 (Cell Signaling Technology, MA, USA), GAPDH (Multisciences, China), 4H7 (Soochow University-Bright Scistar, China), CD276 (R&D Systems, MN, USA), and CXCR4 (EMD Millipore, MA, USA).

The following secondary antibodies were used: horseradish peroxidase-conjugated rabbit anti-goat, goat anti-mouse, and anti-rabbit antibodies (Multisciences, China). Proteins were visualized with an ECL detection kit (Bio-Rad, CA, USA). The following fluorescent secondary antibodies were used: Cy3-conjugated anti-goat IgG (Invitrogen, CA, USA) and Alexa Fluor 633-conjugated anti-rabbit IgG (Invitrogen, CA, USA). The following antibodies were used for flow cytometry: B7-H3-APC (eBiosciences, CA, USA) and CXCR-4-PE-cy7 (eBiosciences, CA, USA).

### Patients and clinical specimens

Tissue samples from 120 gastric cancer patients were analyzed immunohistochemically. Patients were randomly selected from all those undergoing radical gastric cancer resection between 2014 and 2015 at the Department of Pathology of the Second Affiliated Hospital of Soochow University. None had received chemotherapy or radiation therapy before surgery. Gastric carcinoma diagnoses were confirmed via hematoxylin and eosin (H&E) staining after surgical resection. Average patient age was 61.19±1.098 years (range: 28–79 years). The tumor was located in mucosa in 9 cases, in shallow muscularis in 18 cases, and in deep muscularis in 93 cases. Three tumors were well-differentiated, 50 were moderately-differentiated, and 67 were poorly-differentiated (Table 1). This study was approved by the ethics committee of our hospital, and all patients provided written informed consent prior to enrolment.

### Immunohistochemical staining

All surgically resected specimens and biopsy samples were fixed with 10% neutral buffered formalin, embedded in paraffin, and serially sectioned at 4 μm. IHC was performed on selected slides using the ChemMate^TM^ Envision/HRP technique [41]. Briefly, sections were deparaffinized and dehydrated, and endogenous peroxidase activity was blocked using H_2_O_2_. Sections were incubated with B7-H3 and CXCR4 primary antibodies followed by secondary antibody, and visualized with diaminobenzydine (DAB). Finally, slides were counterstained with hematoxylin.

B7-H3 or CXCR4 immunopositivity was evaluated according to overall staining intensity and area (percentage of positively stained tissue). Staining intensity was scored as follows: weak: 1; moderate: 2; strong: 3. Staining area was scored as follows: < 33%: 1; > 33% to <66%: 2; > 66%: 3. Final scores were calculated by multiplying a given case's intensity and area scores. A final grade of < 3 was considered low expression, while > 3 was classified as high. All slides were evaluated independently by two investigators blinded to patient identities and clinical outcomes.

### Gastric cancer cell lines and cultures

The human gastric cancer line, N87, was purchased from ATCC and cultured in RPMI 1640 medium (HyClone, USA) containing 10% heat-inactivated fetal bovine serum (FBS) (Biological Industries, Israel) and 1% penicillin/streptomycin (Biological Industries, Israel). Cells were incubated at 37°C in a humidified chamber with 5% CO_2_.

### shRNA-mediated B7-H3 knockdown in N87 cells

Lentiviral gene transfer vectors encoding green fluorescent protein (GFP) and either a human B7-H3 (NM_001024736, GenBank)-targeting small hairpin RNA (shRNA) (5′-GTGCTGGAGAAAGATCAAA-3′) (LV-B7-H3 virus) or a non-targeting control shRNA (5′-TTCTCCGAACGTGTCACGT-3′) (LV-NC virus) were constructed by Shanghai GeneChem Co. (Shanghai, China). LV-B7-H3 and LV-NC were prepared and titered to 5×10^9^ Tu (transfection units)/ml. N87 cells were subcultured into 6-well tissue culture plates at 5×10^4^ cells/well, and incubated overnight. Virus-containing supernatant was added to cell cultures at a multiplicity of infection (MOI) of 100 with Enhanced Infection Solution (ENi.S) and 5 μg/ml Polybrene. GFP was evaluated by fluorescence microscopy to estimate infection efficiency. After two weeks, cells were selected with 5μg/ml puromycin (Sigma-Aldrich, USA). Antibiotic-resistant clones were isolated and cultured in medium containing 0.5 μg/ml puromycin.

### Flow cytometry

Isolated cells were washed in phosphate-buffered saline (PBS) containing 2% FBS and incubated with fluorochrome-conjugated antibodies (B7-H3-APC or CXCR4-PE-cy7) for 30 min at 4°C. Labeled cells were re-suspended in 0.5 ml cell staining buffer and analyzed using flow cytometry (FlowJo software v 7.6.2, USA). Isotype controls were performed for each staining.

### Western blotting

LV-NC- or LV-B7-H3-infected N87 cells were washed twice with PBS. Cells were incubated in protein lysis buffer containing protease inhibitors for 30 min on ice, and then centrifuged for 30 min at 12,000 rpm at 4°C. Equal amounts (10–30 μg) of total protein extracts were subjected to SDS-PAGE and transferred to PVDF membranes. Membranes were blocked with 5% BSA for 1 h and incubated overnight with primary antibody at 4°C, followed by incubation with the appropriate HRP-conjugated secondary antibody for 2 h at 20°C. Blots were visualized with an ECL detection kit (BIO-RAD, CA, USA) and ChemiScope (Model No. 6300). GAPDH was used as a loading control. Band intensities were calculated via densitometric analysis in Image J (Rawak Software, Inc. Germany).

### Real-time PCR

Total RNA was isolated from LV-NC- or LV-B7-H3-infected N87 cells using TRIzol reagent (Invitrogen, Carlsbad, CA, USA). cDNA was obtained using a reverse transcription reagent kit (Takara, Otsu, Shiga, Japan). Real-time PCR was performed using the Power SYBR Green PCR Master Mix (Takara, Otsu, Shiga, Japan) and products were detected using a gel documentation System (Bio-Rad, CA, USA). Real-time PCR primers used for mRNA quantification were as follows: GAPDH: 5′-AGAAGGTGGGGCTCATTTG-3′and 5′-AGGGGCCATCCACAGTCTTC-3′; B7-H3: 5′-TGT CTCATTGCACTGCTGGT-3′ and 5′-CCTCAGCTCCT GCATTCTCC-3′; and CXCR-4: 5′-GCAGCAGGTAGC AAAGTGAC-3′ and 5′-GAAGTGTATATACTGATC CCCTCCA-3′. Relative genomic expression was calculated via the 2^−ΔΔCt^ method.

### Cell viability assay

Cell proliferation was evaluated using the Cell Counting Kit-8 (CCK-8, Dojindo, Japan) according to the manufacturer's instructions. 0.2×10^4^ LV-NC- or LV-B7-H3-infected N87 cells in 100 μl of RPIM-1640 media supplemented with 10% FBS were seeded into each well of a 96-well plate. At the indicated time points, medium was exchanged for 90 μl RPMI-1640 and 10 μl CCK-8, and cells were incubated for 2 h. Absorbance was measured for each well at a wavelength of 450 nm. Cell growth was monitored every 24 h over 5 d. All experiments were repeated three times.

### Wound healing assay

LV-B7-H3- or LV-NC-infected N87 cells were incubated in 6-well plates and small straight-line wounds were made in confluent monolayers using a 200-μl pipette tip. Cells were washed twice with sterile PBS and incubated in RPMI 1640 medium containing 2% FBS at 37°C in a humidified chamber with 5% CO_2_ for 24 h. Wound images were captured at 0 and 24 h at 100× magnification using an inverted microscope (Leica DM IL LED, Wetzlar, Germany). This experiment was conducted in triplicate.

### Migration and invasion assays

For migration and invasion assays, 0.5×10^4^ cells in 100 μl of serum-free RPMI media were seeded onto the tops of 8-μm pore size transwell chambers or transwell matrigel invasion chambers (BD Biosciences, San Jose, CA, USA). 50 μl matrigel was diluted 1:7 in serum-free media and incubated for 4 h at 37°C in preparation for the transwell matrigel invasion chambers. Lower compartments contained RPMI 1640 medium with 10% FBS. After 24 h incubation at 37°C in a humidified chamber with 5% CO_2_, non-invading cells and gel were removed from the upper chamber using cotton tipped swabs. Cells were fixed with methanol for 30 min and stained with crystal violet. Invading cells were counted in three random fields per filter at 200× magnification for triplicate wells. This experiment was conducted in triplicate.

### Immunofluorescence analyses

IF analysis was used to assess B7-H3 and CXCR4 colocalization in LV-B7-H3- or LV-NC-infected N87 cells. 1 × 10^5^ cells were seeded on glass coverslips in 24-well plates. After 1 d, cells were washed three times in PBS for 5 min/wash, fixed in 4% paraformaldehyde for 5 min, and washed again in PBS three times. Cells were treated with 0.25% Triton X-100 with 0.2% BSA for 5 min and then washed three times in PBS. Cells were blocked with 1% normal horse serum, and incubated with specific primary antibodies (B7-H3 or CXCR4) for 5 h at 4°C. After three PBS washes, cells were incubated with Cy3-conjugated anti-goat IgG (1:400; Jackson ImmunoResearch, USA) or Alexa Fluor 633-conjugated anti-rabbit IgG (1:400; Jackson ImmunoResearch, USA) for 1 h at 37°C, and then rinsed three times with PBS for 5 min/was. Nuclei were counterstained with DAPI (Sigma-Aldrich, USA) for 8 min, and slides were coverslipped. Cells were observed under a laser confocal microscope IX71 (Olympus, Japan) with a digital camera (Olympus, Japan).

### Immunoprecipitation

LV-NC-infected N87 cells were lysed in polysome lysis buffer containing 100mM KCl, 5mM MgCl_2_, 10mM HEPES (pH 7.0), 0.5% NP-40, 1mM DTT, and protease inhibitor cocktail. Lysates were incubated on ice for 30 min, and then centrifuged for 30 min at 12,000 rpm at 4°C to remove cellular debris. Supernatants were added to protein A/G beads (Santa Cruz Biotechnology, CA, USA) with 4H7 (anti-B7-H3 antibody), which was previously incubated overnight at 4°C and was washed five times in wash buffer (50 mM Tris-HCl pH 7.4, 150 mM NaCl, 1 mM MgCl_2_, and 0.05% NP-40). Beads were also washed five times in wash buffer before the bead mixture was incubated at 4°C for 6 h. Proteins were eluted with 2×SDS sample buffer, and then subjected to western blotting analysis.

### Experimental metastasis model in athymic rodents

LV-B7-H3- or LV-NC-infected N87 cells (1×10^6^ in 100 μl PBS) were injected into the tail veins of 4-week-old female BALB/c nude mice. Each group consisted of six mice. Mice were checked daily for symptoms of metastasis, including non-normal physiological conditions suggesting cachexia with weight reduction and weakness. Mice were imaged using an *in vivo* imaging instrument at four weeks to detect GFP^+^ cells in organs. After anesthetization, mice were fixed in position on their backs (PerkinElmer Ivis Spectrum Imaging System). After five weeks, all mice were euthanized. Tissues were removed and fixed in 10% formalin for H&E staining, and metastasized colonies were counted using a dissecting microscope.

### Statistical analysis

The χ^2^ test was performed to compare groups and to evaluate histological and clinical parameter correlations (age, gender, infiltration depth of tumor, lymph nodes metastasis, and degree of differentiation). For *in vitro* experiments, all data were shown as means ± standard deviation (S.D.) of 3 independent experiments, and were analyzed using an unpaired Student's *t*-test. In animal experiments, Kaplan-Meier analysis was used to estimate survival and Gehan-Breslow-Wilcoxon test was used assess differences between the two groups. All statistical analyses were performed using GraphPad Prism 6.0 software (GraphPad Software Inc., San Diego, USA). *P* < 0.05 was considered significant.

## SUPPLEMENTARY MATERIALS FIGURE


