# Supplementary material for: B7-H3 promotes gastric cancer cell migration and invasion
Source: Oncotarget. 2017 May 13;8(42):71725–35. doi: 10.18632/oncotarget.17847 (PMC5641084; doi:10.18632/oncotarget.17847)
Supplement: Supplementary file 1 [file oncotarget-08-71725-s001.pdf]

B7-H3 promotes gastric cancer cell migration and invasion

Supplementary Materials

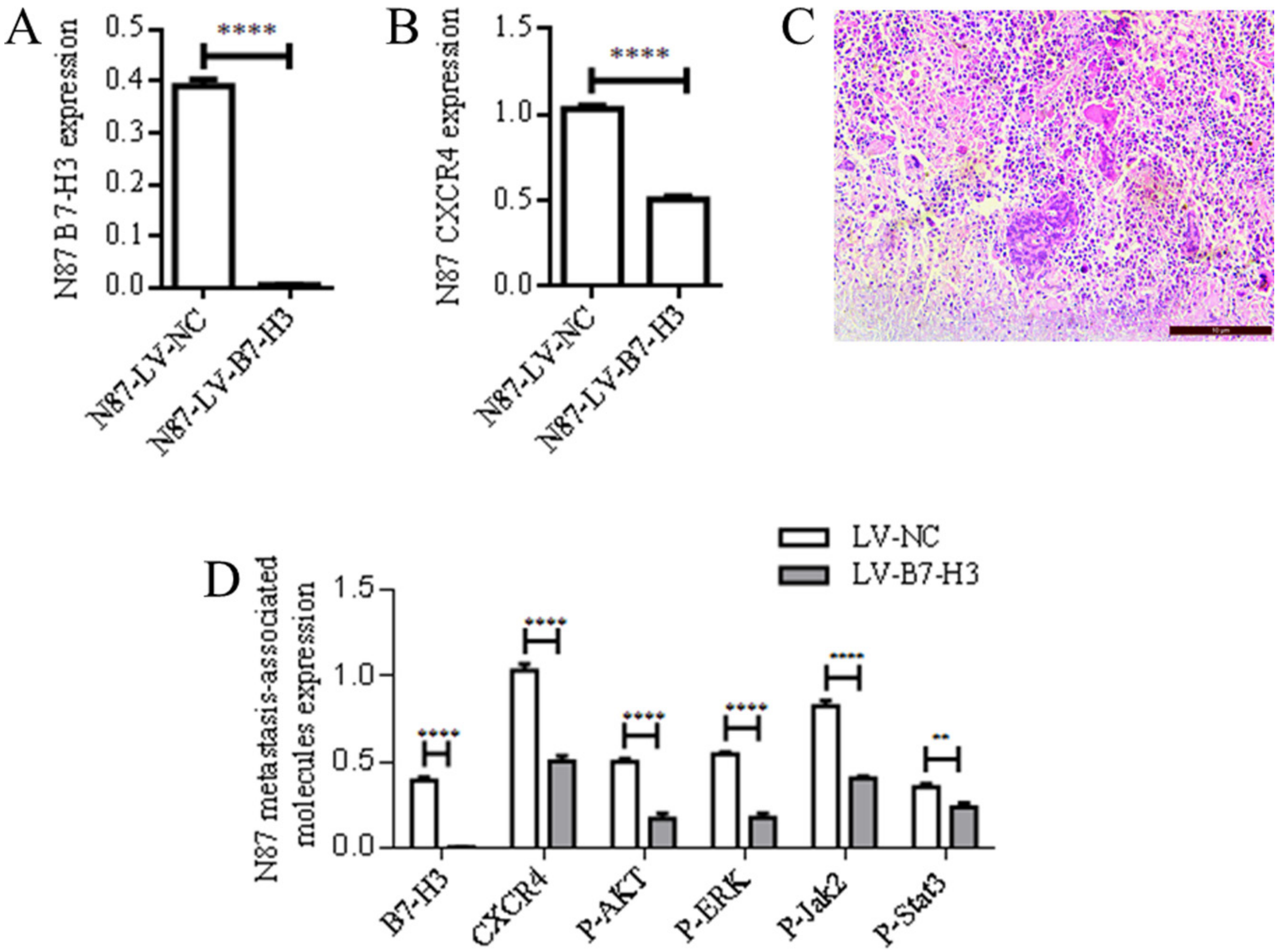

Supplementary Figure 1: Western blotting band densitometric analyses, and spleen metastasis H&E staining. B7-H3 (**A**) and CXCR4 (**B**) plasma protein band densitometric analyses. H&E staining of spleen metastases in mice injected with B7-H3-silenced or control N87 cells (Magnification, ×200) (**C**) Metastasis-associated molecule (B7-H3, CXCR4, p-AKT, p-ERK, p-Jak2, p-Stat3) plasma protein band densitometric analyses (**D**) $*P < 0.005$, $****P < 0.0001$.
